# A qualitative study of Chinese parental perspectives on the causes of Tourette syndrome in children

**DOI:** 10.1038/s41598-024-57062-6

**Published:** 2024-03-18

**Authors:** Yong Hu, Dan Yu, Zheng Liu, Li Zhao, Lingli Zhang, Chunsong Yang

**Affiliations:** 1 Department of Pediatrics, West China Second University Hospital, Sichuan University, Chengdu, China; 2 Key Laboratory of Birth Defects and Related Diseases of Women and Children, Ministry of Education, Sichuan University, Chengdu, 610041 China; 3 Department of Children’s Genetic Endocrinology and Metabolism, West China Second University Hospital, Sichuan University, Chengdu, 610041 China; 4 Department of Pharmacy, West China Second University Hospital, Sichuan University, No. 20, Third Section, Renmin Nan Lu, Chengdu, 610041 Sichuan China; 5 Evidence-Based Pharmacy Center, West China Second University Hospital, Sichuan University, Chengdu, 610041 China; 6 Department of Health Policy and Management, West China School of Public Health/West China Fourth Hospital, Sichuan University, Chengdu, 610041 China

**Keywords:** Causality, Parenting, Perspectives, Qualitative study, Tourette syndrome, Neuronal physiology, Psychology, Neurology

## Abstract

Parents’ beliefs and attitudes toward their children with Tourette syndrome (TS) influence treatment-seeking behaviors. This study aimed to explore and describe the Chinese parents’ perspectives on the causes of TS for their children. A qualitative study using semi-structured interviews was conducted with the parents of TS patients from a children’s hospital in western China from June to July 2021, and thematic analysis was performed to transcribe interviews and identify themes. A total of 13 participants were interviewed in this study. Five themes were developed in relation to the cognition of the causes of TS in parents, including physical problems, parenting and education problems, mental problems, bad habits, and neurological problems. Due to the insufficient awareness of TS, most parents repeatedly seek medical advice that they regarded the symptoms as physical problems or neurological problems. They generally felt guilty and blamed themselves for their parenting styles and education methods. And some parents attributed it to the poor psychological quality or the bad habits of children. Study findings showed a lack of scientific understanding of the causes of TS among parents further hindered the timely effective treatment for patients and affected the family relationships, which highlights the importance of public education and raising awareness of the disease.

## Introduction

Tourette syndrome (TS) is a neurodevelopmental disorder with onset in childhood and adolescence, as one of the most complex and severe subtypes of Tic disorders (TD), characterized by multiple motor tics and vocal tics that last for at least 1 year^1^. It affects 0.52% of the general population and 0.3% to 0.9% of the general pediatric population^2^. Most individuals with TS (85.7%) suffer from one or more lifetime comorbid disorders, such as attention deficit hyperactivity disorder (ADHD), obsessive–compulsive disorder (OCD), anxiety, depression, self-injurious behavior, etc., further increasing the complexity and severity of TS^3^. It seriously affects the individual development and social function of pediatric patients and brings a heavy burden to their families and society. At present, the etiology of TS is still unclear and its pathogenesis could be the result of a combination of genetic, immunological, psychological, and environmental factors^4^.

The management of TS mainly focuses on psychotherapy and pharmacological treatment, and psychoeducation is usually considered as the initial intervention that refers to sharing the latest information about the symptoms, cause, prognosis, treatment, and daily experience of TS with patients and their families^5^. A World Health Organization report states that the beliefs and attitudes held by family members of the community may influence many facets of mental health care, which implies that a favorable social environment contributes to improvement and reintegration while an unfavorable one may encourage stigma against persons with mental illness^6^.

A previous study illustrated the links between parental stress and adverse consequences and exacerbation of the child’s symptomatology due to parents’ perspectives and responses to their kid’s disorder^7^. Parents of children with TS experienced extensive parenting stress and reported lower self-concepts, higher caregiver burden, and marital dysfunction, which limited their caregiver functions and mental health well-being^8–10^. Different scholars stated that parenting stress was associated with greater functional impairment and shame among children with TS which may hinder their recovery from the disorder^11^. It is known that successful treatment and rehabilitation of persons with mental illnesses are influenced by family attitudes and beliefs. Many belief systems held by individuals are based on the preponderant ideas in a society. The previous researcher claimed that general practitioners, teachers, parents, or caregivers of children with TS endorsed the symptoms as bad habits of the children, and they usually comment on their children’s tics and require the children to suppress, and even punish their children for such behaviors which usually worse the children’s symptoms^12^. The misconception about TS leading to increased parenting aggression results in poor family caring function and exacerbated the kids’ TS symptoms^13^.

Besides, the vast range of behaviors associated with TS has often led to the condition being underdiagnosed and treatment due to the parents, general practitioners, and even pediatricians ascribing the symptoms to other conditions, such as nervousness, psychiatric problems, hyperkinesia, behavioral or sleep disorders, etc., or given no formal diagnoses^11^. Obviously, insufficient cognition and misconceptions from professionals, parents, and the public surrounding their child’s symptoms may hinder them from getting timely help^14^. Parents are now viewed as a foundational aspect of mental health systems growth and play an important part in ensuring that persons with physical and psychological disturbances receive systematic care since beliefs and attitudes about causes influence help-seeking behavior and treatment. The impact of TS on the parents of those diagnosed, however, has not been thoroughly investigated in China. Thus, in this study, we set out to examine and identify the Chinese parents’ perspectives on the causes of TS in their children to discover the influence on their treatment-seeking behaviors.

## Materials and methods

### Study design

A qualitative research using semi-structured in-depth interviews was performed to understand the parental cognition of the causes of tic and their personal feelings about the disease through the personal narration of the parents.

### Participants and settings

The research was conducted at West China Second University Hospital, Sichuan University from June to July 2021. Purposive sampling was used to select interviewees meeting the inclusion criteria that: (i) patients with a definitive diagnosis of TS, the diagnostic criteria refer to China clinical practice guidelines for TS and ADHD^15,16^. (ii) patients’ parents with comprehension and communication capability. The exclusion criterion was patients with exhibited cerebral palsy, meningitis, motor language development lags, nail-biting, restless legs syndrome, myasthenia gravis, Brown syndrome, or other neuropsychiatric conditions. We approached potentially eligible parents volunteering to participate in this study, of which were finally included in consideration of the strategic orientations based on their gender, age, severity of disease, parents’ education background, job, and social background, history of neurodevelopmental and psychiatric disease, for increasing their representativeness. We collected the information of family history of TD, family history of other neurodevelopmental and psychiatric disorders, the Yale Global Tic Severity Scale (YGTSS) scores, and medication. YGTSS was used to assess current tic severity, which yields 3 summary scores: total motor scores (0–25), total phonic scores (0–25), and impairment scores (0–50), which evaluates the global level of functional impairment arising from tics^17^.

### Data collection

All the research was conducted in quiet rooms in the hospital, where participants were interviewed for 25–50 min (with an average of 36 min). Before the interview commenced, the purpose and method of the interview were introduced by two interviewers who had received qualitative research training. After obtaining participants’ written informed consent, the interview was audio-recorded and began with open-ended questions. The participants were invited to talk about their cognition of TS and personal understanding and experience of the causes of the disease. During the interview, the interviewers also asked some probing questions based on the answers. When theoretical saturation was achieved, interviews with new questions were stopped, which was measured from the perspectives of participants and researchers. From the interviewee’s point of view, if the interviewee has finished speaking or has no relevant content to express, then the information is saturated for the interviewee. From the researcher’s point of view, if the researcher thinks that the information obtained has reached the two extremes of his cognition of a certain problem, or is enough to analyze his research goal, then the information is saturated^18^. All data collection was both private and anonymous.

### Data analysis

Two researchers independently conducted the thematic analysis of the interviews. After being transcribed into Word documents, all the interview records were transferred to Excel for coding. The Braun and Clarke’s six-phase method^19^ was used to guide qualitative data analysis, including the following steps: (i) getting familiar with the interview content and developing a basic perception of the interview content; (ii) generating the initial coding according to the interview content; (iii) searching for the themes, which was, to find the common core themes based on the initial coding through repeated comparison; (iv) reviewing, revising and refining the themes obtained in the early stage; (v) defining and naming the themes to obtain more refined core themes, which were consistent with the interview data based on the early steps; (vi) writing the report.

### Ethical approval

This study has been approved by the Ethics Committee of West China Second University Hospital, Sichuan University. Each patient and their parents have provided informed consent in writing, and all participants were informed that the interview content and the recording were confidential and anonymous. Research involving human research participants must have been performed in accordance with the Declaration of Helsinki. Written informed consent was obtained before the interview from all subjects involved in the study.

## Results

### Characteristics of participants

In total, 13 parents received the interview in this study, including 4 males and 9 females, with a mean age of 36.4 years old (range 29–50 years old). The mean age of their children with TS was 7.8 years old (range 4–11 years old), including 10 boys and 3 girls, among which 2 children had reported comorbid disorders. On average, the duration of TS in pediatric patients was 2.4 years (range 1.0–5.0 years). The demographic information of participants was shown in Tables 1 and 2.

**Table 1:** Characteristics of participants.

No	Parent				
	Gender	Age	Resident	Education background	Neurodevelopmental and psychiatric disease
P1	Female	34	Urban	College	No
P2	Female	50	Urban	Secondary school	Yes
P3	Female	39	Urban	College	No
P4	Female	46	Rural	College	No
P5	Male	38	Urban	Secondary school	No
P6	Male	33	Urban	Secondary school	No
P7	Female	32	Urban	Secondary school	No
P8	Female	37	Urban	College	No
P9	Male	32	Urban	College	No
P10	Female	34	Urban	College	No
P11	Female	29	Suburban	College	No
P12	Male	34	Urban	College	No
P13	Female	35	Rural	Secondary school	No

**Table 2:** Characteristics of Patients.

No	Gender	Age	Duration of TS (year)	Newly diagnosed cases	Comorbid disorder	Family history of TD	Family history of other neurodevelopmental and psychiatric disorders	YGTSS			Medications
								Total motor scores (0–25)	Total phonic scores (0–25)	Impairment scores (0–50)	
C1	Boy	6	1.2	No	No	No	No	11	4	10	Tiapride
C2	Boy	10	2.5	No	No	Yes	No	15	6	10	Clonidine
C3	Girl	6	2.2	No	No	No	No	8	8	0	Tiapride
C4	Boy	11	5.0	No	No	No	No	10	14	20	Tiapride
C5	Girl	4	1.0	Yes	No	No	No	9	12	0	Clonidine
C6	Boy	11	5.0	No	No	No	No	10	10	10	Tiapride
C7	Boy	11	2.5	No	Yes, ADHD	No	No	10	14	20	Tiapride + Clonidine
C8	Boy	7	2.4	No	No	No	No	6	10	10	Tiapride
C9	Girl	5	1.0	Yes	No	No	No	12	8	0	Tiapride
C10	Boy	8	1.8	No	No	No	No	11	10	10	Tiapride
C11	Boy	5	2.5	No	No	No	No	7	12	10	Tiapride
C12	Boy	7	2.0	No	Yes, ADHD	No	No	13	8	10	Tiapride + Clonidine
C13	Boy	10	1.5	No	No	No	Yes	10	12	10	Tiapride

### Physical problems: repeated seeking medical advice

Because the main clinical manifestations of patients with TS included blinking eyes, making noise, twisting the neck, etc., most parents would repeatedly take their children to the ophthalmology department, otolaryngology department, internal medicine department, etc. in different hospitals.

The child blinked at first, and then we took him to the ophthalmology department but there was nothing wrong with the eyes. The doctor prescribed eye drops for him, which worked at that time. However, the second time we went to the hospital, the same eye drops did not work. Then the child’s symptoms were relieved and he didn’t blink anymore. But after a time, he began to blink again…. He often caught colds when he was young. After the cold, he would always cough and feel uncomfortable in his throat. At that time, we thought the cold may not be completely cured, so we went to see a doctor for it again. But he felt that there was still something wrong about his throat. (P1)

Another parent expressed a similar statement. Many parents lack cognition of the disease.

At the beginning, I thought the problems were about his eyes, then the throat, and finally we found it was TS…. When we figured out the disease we started to pay attention to the disease. It was really hard to identify because we had little cognition about the disease. (P11)

Other parents even repeatedly visited the hospital, but they could not get a definitive diagnosis and treat the disease.

The disease had lasted for over one year. The doctors first prescribed some medicine and told us to keep an eye on his condition. But the child didn’t have any symptoms for more than half one year. From last month, he nodded at times, and I suggested to go to the hospital so that he could have an electroencephalogram examination, but failed because the doctor didn’t suggest we do so. (P10)

It can be concluded that due to the lack of systematic cognition of the disease among parents, nurturers, and doctors, the patients did not receive effective treatment in time, which delayed their condition and caused a lot of trouble and pain to the patients and their parents.

### Parenting and education problems: guilt and self-blame

Some parents often attributed the causes of disease to the strict education methods, either from their own or from society. As a result, parents gave up the management of their children’s behavior or overcompensated their children. They even took their children to seek medical treatment repeatedly aiming to eliminate any symptoms to make up for their guilt.

The main reason might be that I was too strict with my son. Since childhood, he didn’t listen to anyone in our family but me. As long as I was at home, he would always obey what I said. So the cause of his disease might be that I was so strict with him that he always underwent a lot of stress. Despite that, we could not find another reason for the disease. (P8)

This parent felt very guilty about her education methods and wanted to find the best doctor to completely cure her child. Undoubtedly, the family members’ feeling of guilt and self-blame also affected their medical-seeking behavior, making them have unrealistic expectations for the treatment.

Last semester he had a new teacher who was also a strict person requiring good academic performance. Although it was great, it also put much pressure on the children. When my kid didn’t finish his homework or felt it was difficult to do, the teacher would ask him to his office or even ask parents to watch children finish their homework, which also put pressure on the parents. Sometimes I lost control of my temper and got mad at my child. This semester he had another new teacher, and he got much better at his disease…. Now I am trying my best to satisfy his need. (P7)

Parenting problems made parents feel guilty, thus they constantly sought treatment to fix the problems, gave up the requirements for children, or excessively satisfied their children. Most parents were just trying to relieve their feelings of guilt or anxiety and satisfy their wish to be good parents.

### Mental problems: worries and fears

When a child was sick, many parents habitually thought the disease was because of mental stress, cowardice, or other psychological factors.

Well, my son had been sleeping with us since he was born. After he was six years old, he began to sleep in a separate room. Since then, he slept at night with the light on and never closed the door because he was afraid of the dark. I thought this had a certain relationship with his psychology. I have been thinking about this problem, and finally, in order to confirm my guess, I came to your hospital to find a doctor to give him psychological counseling. (P6)

Many parents believed that the child’s symptoms would be relieved if their mental problems were settled.

I thought it had some reasons from external impacts, for example, the way I brought him up. To be honest, I didn’t stay with him every day until he was seven years old and began to enter primary school. Before then, he grew up with his grandmother. What I’m trying to do right now is to open his heart and make him more extroverted. My husband and I once got him a psychologist who gave him some help. (P2)

Some parents excessively or irrationally thought that psychological factors determined the symptoms of patients, and they would continue to provide psychological services to patients while ignoring the complexity of the disease, resulting in discontinuation of medicine in the treatment.

### Bad habits: keep correcting

Some parents, lacking a systematic understanding of the disease, often blamed their children for their bad habits. Many parents had similar statements.

Such as blinking, I think she got into the habit of blinking because she always did so... I didn’t even think it as a disease. (P3)

Another parent even blamed the patient for watching TV and mobile phone for a long time and attributed these to the child’s behavior problems. Due to the uncontrollability and impulsiveness of the tic, it undoubtedly aggravated the children’s anxiety and self-blame.

My child relapsed two years after he was cured. Is it related to his playing mobile phones and staying up late for a long time, that is, staying up all night or at the weekend? Because he relapsed during Chinese New Year when people liked to play in rural areas. It didn’t happen anything before that year, but he got sick after when he played with other children, watched the phone or TV, and stayed up late for 5 or 6 nights. (P4)

Other parents even punished their children because they thought the disease was a habit problem, which worsened the family relationship and their symptoms.

I was very angry because of these bad habits. In that period, especially when he was eight years old, he developed those bad habits. I was so angry that I beat him several times. For a few times, I beat him so hard that his skin was broken. (P13)

Almost all parents claimed that at the beginning they thought it was a matter of habits, and asked patients to control their symptoms of tic. This unreasonable way of parenting certainly could not effectively help patients and would increase their sense of losing control and the conflicts between requirements from parents and the uncontrolled tic.

### Neurological problems: misunderstanding of tics

In many cases, the patient’s parents had their understanding of the doctor’s diagnosis. For example, they thought tics meant “pumping” and “moving”. “Pumping” was usually regarded as convulsion or epilepsy. “Moving” was usually regarded as hyperactivity, that is, ADHD. Such misunderstandings undoubtedly affected patients’ medical treatment.

At that time, I thought he watched TV too often so I forbade him for a time, and then he got better. I never thought that he actually had ADHD. (P12)

Because many parents misunderstand the literal meaning of tics and their children’s physical movements, they regarded this disease as ADHD even though doctors had told parents the truth.

Other parents had similar perceptions and beliefs. According to the traditional cognition in China, nervous system diseases are difficult to treat and would affect the intelligence of patients. Thus, many parents are sensitive and afraid after hearing the diagnosis.

That was exactly what I worried about. I’ve been worrying about her syndrome, the nervous system disease, and whether I would also have such a disease. It made me insomnia and woke up at night over and over again. (P9)

Since in Chinese words, literally “tic” was very similar to “convulsion”, which was equal to epilepsy. These misunderstandings affected the motivation in the family and increased the parents’ sense of powerlessness, anxiety, and fear.

I’m afraid that she had convulsions… that means epilepsy, and it feels like she had a seizure. I’m worried this disease can’t be cured. (P5)

It is very important to help parents have a reasonable understanding of the disease and explain it in an easy way that they can understand. Meanwhile, medical workers should not only give a diagnosis but also clarify the understanding of patients and their families on the diagnosis. Constant clarification is needed in doctor-patient communication.

## Discussion

To the best of our knowledge, this is the first study in China addressing the parents of children with TS. Previous studies have examined the perspectives on the causes of TS among community members in other nations. It is very important for parents of children with TS to understand and accept the complexity of the disorder which involves both genetic and environmental factors. Our results reveal a prevalent belief in physical-psychological-educational-developmental-habitat causes among caregivers.

One key factor affecting the cognition of the impairment of TS in China is the commonly held belief among parents that TS represents bad habits, rather than physical symptoms. The throat clearing, coughing, other sounds with the mouth, and other TS symptoms repeatedly presented by the kids are treated as bad habits. Tics were thought to be under voluntary control which was usually required to suppress by parents. If the children cannot suppress the involuntary behavior, they may experience an increasing loss of control over tics and increasing anxiety-related symptoms. As mentioned by Leckman and his colleagues, if the involuntary character was misunderstood as voluntary which could be a burdensome source of anguish and guilt for kids with TS, they will question themselves about why they cannot control and what is wrong with themselves^20^. Besides, the misunderstanding of TS can lead to conflict with family members, as they accuse the young person of deliberately doing it or not trying enough to stop the tic, which can have a profound impact on the young person’s ability to make sense of his/her inner experiences and increase their sense of shame toward the involuntary behavior. As we know, TS is a neurological syndrome that all of the tics are involuntary. To inform the parents that the characteristic of involuntary TS can reduce inappropriate parenting and suppress behaviors toward the children.

Many parents claimed that they bring their children to different clinicians, and they are diagnosed with different physical diseases such as eye problems, respiratory problems, throat problems, etc., Research and clinical experience suggest that, in the majority of cases, both children and their parents indicate that the most disabling symptoms relate to disruptive behavior, hyperactivity, and temper outbursts, rather than tics^21,22^. Consistent with the previous study^23^, our study implies that health professionals have limited knowledge and consciousness of TS, which limits patients to get the effective referral, treatment, or timely health services.

As discussed in Malli’s study, parents expressed guilt about their children’s condition^24^. This study discovers that the parents feel guilty or self-blame in relation to their beliefs that their education failure or emotional instability contribute to the causes of TS in their children. Parents also experience the loss of their ‘ideal child’ due to their dysfunction which is also deeply distressing^25^. Guilt feelings of parents usually impair the parent–child and sibling relationships and further elevate levels of overall family stress^26^.

Similarly, this study reveals that parents still simply endorse psychogenic causes and seek help or psychotherapy approaches to tackle the disorder. This suggests that endorsing psychogenic causes usually influences their help-seeking behaviors. The previous researcher also claimed that parents believed psychogenic treatment may hinder them from accepting or endorsing multiple causes of the disorder^27^. Belief in psychogenic causes may result in a tendency to underestimate medical treatment due to the belief that psychotherapy is more fundamental.

There are also likely to be important differences in understanding and recognition of symptoms between different cultures^28^. Tic is translated into Chinese as “Chou Dong”. Chou literately means convulsion, which is usually misunderstood as epilepsy. Dong literately means movement and is misunderstood as the disease of ADHD among some parents. Meanwhile, the disorder of TS is usually comorbid with ADHD. We discover that the conceptualization and understanding of the disorder are different due to individual reprocessing of the literal meaning, potentially affecting illness experiences and medical-seeking behaviors.

This study has some limitations. Firstly, this study examined causal attribution in parents of patients who attended the outpatient department of a children’s hospital in western China, so care should be taken when generalizing the results from this study to other areas. Second, the study did not explore different caregivers’ perspectives of the disease, which may not catch a comprehensive picture of how different caregivers view the symptoms.

The results of this study reveal that participants lack of scientific understanding of causal attributions for TS. Those with no formal understanding all embraced habit, psychogenic, educational, and other environmental causes. This suggests that it is emergent to provide popularization which may impact or change perspectives on the causes. The education and enlightenment campaigns have a role to play in changing beliefs and consequently, attitudes to TS, while it is also important to improve access to treatment to ensure that treatment can be received as early as possible. Besides, it is pivotal to train general health practitioners to be more conscious of TS which can help the patients get timely referrals and access to effective medical services.

## Data Availability

The data used to support the findings of this study are available from the corresponding authors upon reasonable request.

## Abbreviations

TS: Tourette syndrome
TD: Tic disorders
ADHD: Attention deficit hyperactivity disorder
OCD: Obsessive-compulsive disorder
